# Protocol for a two-arm pragmatic stepped-wedge hybrid effectiveness-implementation trial evaluating Engagement and Collaborative Management to Proactively Advance Sepsis Survivorship (ENCOMPASS)

**DOI:** 10.1186/s12913-021-06521-1

**Published:** 2021-06-02

**Authors:** Marc Kowalkowski, Tara Eaton, Andrew McWilliams, Hazel Tapp, Aleta Rios, Stephanie Murphy, Ryan Burns, Bella Gutnik, Katherine O’Hare, Lewis McCurdy, Michael Dulin, Christopher Blanchette, Shih-Hsiung Chou, Scott Halpern, Derek C. Angus, Stephanie P. Taylor

**Affiliations:** 1 Center for Outcomes Research and Evaluation, Atrium Health, 1300 Scott Ave, Charlotte, NC 28203 USA; 2 Department of Internal Medicine, Atrium Health, Charlotte, USA; 3 Department of Family Medicine, Atrium Health, Charlotte, USA; 4 Ambulatory Care Management, Atrium Health, Charlotte, USA; 5 Division of Infectious Disease, Department of Internal Medicine, Atrium Health, Charlotte, USA; 6 Academy for Population Health Innovation, University of North Carolina Charlotte & Mecklenburg County Public Health Department, Charlotte, USA; 7 Department of Public Health Sciences, University of North Carolina Charlotte, Charlotte, USA; 8 Health Economics and Outcomes Research Strategy, Novo Nordisk, Plainsboro Township, USA; 9 Palliative and Advanced Illness Research (PAIR) Center, Perelman School of Medicine, University of Pennsylvania, Philadelphia, USA; 10 Department of Medicine, Perelman School of Medicine, University of Pennsylvania, Philadelphia, USA; 11 Clinical Research, Investigation, and Systems Modeling of Acute illness (CRISMA) Center, University of Pittsburgh, Pittsburgh, USA; 12 Department of Critical Care Medicine, University of Pittsburgh, Pittsburgh, USA

**Keywords:** Sepsis, Infection, Continuity of patient care, Patient navigator, Health services, Pragmatic clinical trial

## Abstract

**Background:**

Sepsis survivors experience high morbidity and mortality, and healthcare systems lack effective strategies to address patient needs after hospital discharge. The Sepsis Transition and Recovery (STAR) program is a navigator-led, telehealth-based multicomponent strategy to provide proactive care coordination and monitoring of high-risk patients using evidence-driven, post-sepsis care tasks. The purpose of this study is to evaluate the effectiveness of STAR to improve outcomes for sepsis patients and to examine contextual factors that influence STAR implementation.

**Methods:**

This study uses a hybrid type I effectiveness-implementation design to concurrently test clinical effectiveness and gather implementation data. The effectiveness evaluation is a two-arm, pragmatic, stepped-wedge cluster randomized controlled trial at eight hospitals in North Carolina comparing clinical outcomes between sepsis survivors who receive Usual Care versus care delivered through STAR. Each hospital begins in a Usual Care control phase and transitions to STAR in a randomly assigned sequence (one every 4 months). During months that a hospital is allocated to Usual Care, all eligible patients will receive usual care. Once a hospital transitions to STAR, all eligible patients will receive STAR during their hospitalization and extending through 90 days from discharge. STAR includes centrally located nurse navigators using telephonic counseling and electronic health record-based support to facilitate best-practice post-sepsis care strategies including post-discharge review of medications, evaluation for new impairments or symptoms, monitoring existing comorbidities, and palliative care referral when appropriate. Adults admitted with suspected sepsis, defined by clinical criteria for infection and organ failure, are included. Planned enrollment is 4032 patients during a 36-month period. The primary effectiveness outcome is the composite of all-cause hospital readmission or mortality within 90 days of discharge. A mixed-methods implementation evaluation will be conducted before, during, and after STAR implementation.

**Discussion:**

This pragmatic evaluation will test the effectiveness of STAR to reduce combined hospital readmissions and mortality, while identifying key implementation factors. Results will provide practical information to advance understanding of how to integrate post-sepsis management across care settings and facilitate implementation, dissemination, and sustained utilization of best-practice post-sepsis management strategies in other heterogeneous healthcare delivery systems.

**Trial registration:**

NCT04495946. Submitted July 7, 2020; Posted August 3, 2020.

**Supplementary Information:**

The online version contains supplementary material available at 10.1186/s12913-021-06521-1.

Contributions to the literature

- Sepsis survivors experience high morbidity and mortality, and healthcare systems lack effective strategies to address patient needs after hospital discharge.
- The STAR program extends nurse navigator support during post-sepsis care to facilitate targeted, evidence-driven recommendations using an integrated, patient-centric telehealth approach.
- The proposed study combines a pragmatic randomized trial framework with longitudinal implementation science and prospective economic evaluations to compare usual care versus care delivered with added STAR program support for improving post-sepsis care and outcomes.
- Research will add new practical information to advance understanding of how to best integrate effective post-sepsis management across care settings and heterogeneous healthcare systems.

## Background

Sepsis is a common, life-threatening condition defined by organ dysfunction due to a dysregulated response to infection [1]. Recent policy initiatives have helped to improve timely sepsis identification and treatment and decrease hospital mortality for patients hospitalized with sepsis [2–4]. However, sepsis survivors continue to face challenges after the acute illness episode and experience poor long-term outcomes, including new functional, cognitive, and psychological deficits, and high rates of hospital readmission and mortality in the 90-days after hospital discharge [4–11].

We developed the Sepsis Transition and Recovery (STAR) program to address persistent morbidity and mortality for sepsis survivors. The STAR program uses telehealth nurse navigation to deliver a bundle of best-practice care elements for longitudinal post-sepsis care up to 90 days. These care elements are directed towards the specific challenges and sequelae following a sepsis hospitalization and include: 1) identification and treatment of new physical, mental, and cognitive deficits; 2) review and adjustment of medications; 3) surveillance of treatable conditions that commonly lead to poor outcomes including chronic conditions that may de-stabilize during sepsis and recovery; and 4) focus on palliative care when appropriate [12]. Prior observational data have shown these care elements to be associated with improved outcomes for sepsis survivors [13]. However, they are not widely applied in real-world settings for this vulnerable population, likely hindered by a gap in understanding key contextual factors underlying how to best integrate this bundle of care elements into the complex and fragmented post-discharge setting [14–19].

The objectives of the Engagement and Collaborative Management to Proactively Advance Sepsis Survivorship (ENCOMPASS) trial are to concurrently evaluate: i) the effectiveness of the STAR intervention for reducing mortality and hospital readmission assessed 90 days after hospitalization among high-risk sepsis survivors, and ii) contextual factors related to the real-world implementation of the program. Furthermore, because implementing support programs such as STAR requires health system investments, we will also evaluate the effect of these investments on costs and cost-effectiveness.

## Methods

### Study design

**Fig. 1:**
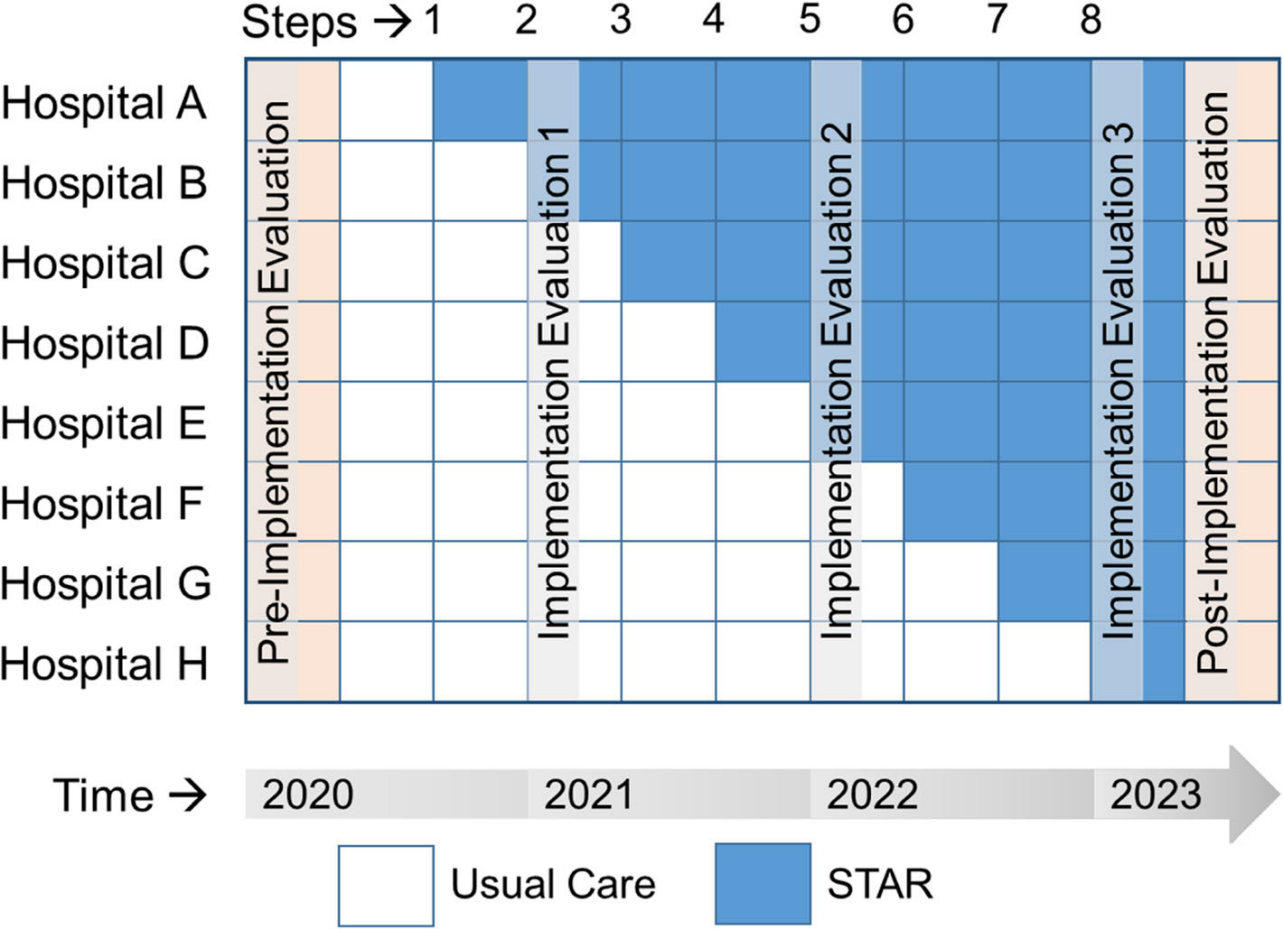
Overview of ENCOMPASS Stepped-Wedge Trial Design and Timeline. The ENCOMPASS trial design is depicted. Each study hospital begins in the Usual Care condition. Every 4 months, one study hospital transitions from Usual Care to the Sepsis Transition and Recovery (STAR) Program for the remainder of the trial. The sequence and timing of the transition for each hospital is randomly assigned. The total ENCOMPASS trial enrollment interval is 36 months. Implementation is evaluated before, during, and after the patient enrollment interval

ENCOMPASS is an effectiveness-implementation hybrid type I trial. The evaluation component is designed as a two-arm, pragmatic, stepped-wedge cluster randomized controlled trial conducted at eight hospitals in which each participating hospital begins in a usual care control phase and transitions to the STAR program intervention in a randomly assigned sequence, with one of eight hospitals assigned to transition at each four-month interval (i.e., step; Fig. 1). Participant enrollment and randomization will take place from July 2020 through June 2023. During the time that a hospital is allocated to usual care, all eligible patients will receive usual care. Once a hospital has been allocated to the STAR arm, all eligible patients will receive STAR during their index hospitalization and extending through 90 days from discharge or date of death. We will capture patient eligibility and outcomes directly from routinely collected data housed in the EHR system and data warehouse. Primary and secondary clinical outcomes will be assessed 90 days after discharge from index hospitalization. We will also use a mixed methods approach to examine implementation of STAR and inform best practices for future dissemination of transition services for sepsis patients.

The hybrid type I trial design combining comparative effectiveness evaluation with collection of implementation outcomes is optimal for ENCOMPASS because STAR satisfies previously described design considerations [20], including 1) there is strong face validity for the recommended post-sepsis care elements and extends existing evidence for delivery of successful care transitions interventions leveraging nurse navigation; 2) indirect evidence supports an association between delivery of the care elements and reduced readmission and mortality [13]; 3) the intervention is associated with minimal risk; and 4) there is “implementation momentum” for the adoption of focused transition support services for high-risk patients [21, 22]. We selected a stepped-wedge randomized design for ENCOMPASS, rather than patient-level randomization, because the intervention is delivered at the hospital level and individual patient randomization could potentiate contamination. The stepped-wedge design also has practical advantages over traditional parallel-cluster randomized designs because it allows for staggered implementation of the intervention across all hospitals, thereby promoting hospital enthusiasm for participating and providing time to prepare each hospital for deployment of the intervention [23, 24]. This trial was approved by the Advarra Institutional Review Board (IRB) with a waiver of informed consent as this evaluation utilizes elements routinely collected in usual clinical practice and deemed to present minimal risk to study participants (IRB#Pro00036873 v1.0 June 26, 2019). The trial is registered with ClinicalTrials.gov (NCT#04495946), and the trial protocol adheres to the Standardized Protocol Items: Recommendations for Interventional Trials (SPIRIT) guidelines [25] (Additional File), the CONSORT extension for cluster randomized trials [26], and the Pragmatic Explanatory Continuum Indicator Summary 2 (PRECIS-2) domains for the design of pragmatic studies [27].

### Conceptual framework

**Fig. 2:**
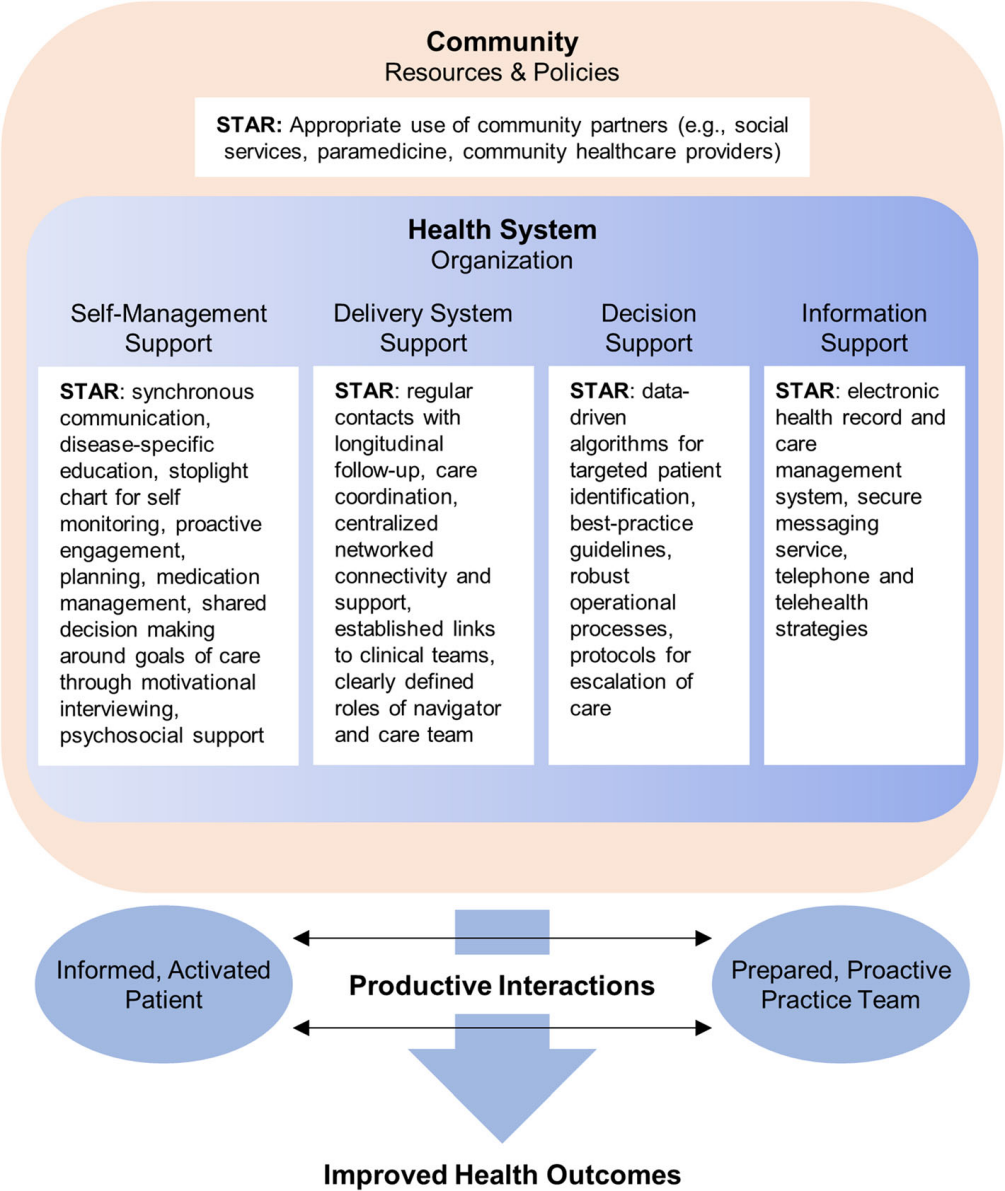
Conceptual model describing the integration of STAR to improve post-sepsis care and outcomes. The elements of the Sepsis Transition and Recovery (STAR) program are shown mapped onto the Chronic Care Model framework. Adapted from *Wagner EH, Chronic disease management: what will it take to improve care for chronic illness? Eff Clin Pract. 1998*

We developed the STAR program to enhance the transition process for sepsis survivors based on the widely-adopted Chronic Care Model (Fig. 2) [28, 29]. The aim of the Chronic Care Model is to transform patient care from acute and reactive to proactive and planned via six interrelated system changes intended to promote productive interactions between an informed, activated patient and prepared, proactive care team. In STAR, nurse navigators are trained to provide disease and health system education, help patients overcome medical system barriers to recommended care, and bridge gaps in service that can serve as points of failure for complex sepsis patients [30]. Additionally, navigators function to address insurance and other financial barriers, aid in care coordination, increase appropriate referrals to community resources, and use motivational interviewing to encourage behavior change, increase proactive patient-provider interactions and improve outcomes [31–34].

The implementation determinants evaluation in ENCOMPASS is conceptually guided by the Consolidated Framework for Implementation Research (CFIR) [35, 36]. We will use the five CFIR domains (intervention characteristics, outer setting, inner setting, characteristics of individuals, and process) to guide planning, organization, and conduct of STAR implementation and to evaluate and adapt the implementation strategy, observe fidelity, and examine sustainability and potential for wider dissemination. The breadth of constructs included within the CFIR enables our study to inform how health systems effectively implement interventions that improve outcomes for sepsis survivors across heterogenous settings and diverse populations.

### Study setting

We will test the STAR program intervention within the course of providing usual care among a large and diverse population of post-sepsis patients admitted to eight hospitals within Atrium Health (AH), one of the largest, vertically integrated health systems in the US. The eight acute care hospitals participating in this study use the same EHR, which connects across all points of care, including outpatient practices, urgent care locations, emergency departments and hospitals. Additionally, study hospitals vary in size and provide care to diverse regions of North Carolina, representing seven different counties (Table 1). We deliberately selected non-tertiary, geographically diverse acute care hospitals for our study to demonstrate the scalability and generalizability of STAR. Specifically, over one-half of sepsis patients in the US receive care at hospitals that have fewer than 500 beds [37, 38]. Our selection of comparably sized hospitals reflects the importance of broadly disseminating successful implementation strategies to non-tertiary facilities providing sepsis care. The diversity of clinical care, established telehealth capabilities, and integrated EHR platform make Atrium Health an ideal environment for this pragmatic trial to evaluate STAR program implementation across geographically, culturally, and economically diverse settings.

**Table 1:** Characteristics of ENCOMPASS participant hospitals

Hospital	Location	Setting	IP / ICU Beds
CHS Blue Ridge	Morganton, NC	Rural	269 / 16
AH Cleveland	Shelby, NC	Rural	241 / 18
AH Pineville	Charlotte, NC	Urban, community	235 / 30
AH Union	Monroe, NC	Suburban, community	175 / 14
AH Stanly	Albemarle, NC	Rural	109 / 10
AH Lincoln	Lincolnton, NC	Rural	101 / 10
AH University City	Charlotte, NC	Urban, community	100 / 8
AH Kings Mountain	Kings Mountain, NC	Rural	67 / 6

*CHS* Carolinas Healthcare System, *AH* Atrium Health, *IP* Inpatient, *ICU* Intensive Care Unit

### Identification and recruitment of eligible sepsis patients

Prior to the initiation of recruitment for ENCOMPASS, the study team worked with key stakeholders to define sepsis patients with high post-sepsis care needs likely to benefit from the STAR program, based on clinical sepsis criteria and coexisting risk profiles [39]. Consistent with our pragmatic study design concept, eligibility criteria are broad, the sample size is large and diverse, and study procedures are embedded into the context of routine care. Patients are eligible if they present to the emergency department and are subsequently admitted under inpatient or observation status to a participating hospital and meet the following criteria: 1) ≥18 years of age upon admission; 2) clinically suspected infection (i.e., antibiotic and bacterial culture orders and two or more markers of systemic inflammatory response syndrome [40, 41] within 24 h of presentation); 3) organ dysfunction, defined as two or more points on either admission Sequential Organ Failure Assessment (SOFA) or quick-SOFA scores [42–45]; 4) deemed to be at high risk of hospital readmission within 90 days, defined as a readmission risk probability ≥ 25% using a previously validated model described below; and 5) not discharged from the hospital at the time the daily list of eligible patients is generated each weekday morning.

We will exclude patients: 1) with a change in code status (i.e., initially full code followed by change to do not resuscitate and/or do not intubate) within 24 h after index presentation due to presumed limitation of aggressive treatment and exposure to STAR program components; 2) reside > 2.5 h drive time from the treating hospital due to the maximum reach of the community services leveraged within the STAR program and the general assumption that these patients may have less comprehensive utilization tracking within AH record systems for study outcomes; 3) are actively participating in a different AH care management program (e.g., cancer care patient navigation) documented in the EHR at time of hospital admission; or 4) have been previously randomized as part of this study.

Our readmission risk model includes clinical and administrative data selected based on their association with post-sepsis outcomes and conceptual models of post-sepsis pathophysiology such as: physiologic measurements, lab values, basic sociodemographic characteristics, and personal medical history [46–48]. All model covariates are sourced from a patient’s routinely-captured clinical data during hospitalization and billing history at the time of hospital admission to produce a near-real time risk score that identifies cohorts at high risk for 90-day hospital readmission. The EHR-based algorithm will run each morning and automatically generate a list of newly eligible, high-risk patients admitted over the prior 72 h. Because we will use a sensitive “suspected sepsis” definition to enroll eligible high-risk patients in near real time, we validated our algorithm against an ultimate determination of sepsis at hospital discharge prior to ENCOMPASS trial recruitment. Our blinded review of 100 consecutively identified patients across the eight participating hospitals found that 55 patients were deemed to have sepsis (i.e., infection with organ dysfunction) at discharge. Using chart review as the reference standard, our EHR algorithm had 93% sensitivity and 80% specificity.

### Study processes

For patients in both arms, data from the automated, daily patient list are sent electronically to the study database. During the intervention phase at each facility (i.e., after transition from usual care), the navigator receives the list of admitted, eligible high-risk patients via secure email. The navigator contacts eligible patients at intervention hospitals by telephone and introduces the STAR process before discharge. Our team has successfully deployed these same processes during prior transitions interventions [49]. In this real-world implementation study, patients, clinicians, and investigators are not blinded to group assignments; but, primary outcomes are collected from objective data, minimizing bias introduced by the lack of blinding. At any point, patients may decline participation in STAR or any components of usual care.

### Trial interventions

Arm 1: Usual Care. When assigned to Usual Care, hospitals and their providers will not have access to the STAR program. Patients in the Usual Care group continue to receive routine clinical care throughout their hospitalization and following hospital discharge. Usual Care elements are not prescribed but, in everyday practice, typically consist of: patient education and follow-up instructions at discharge; routine recommendations for timely outpatient follow-up visits; arrangements for home health services, use of existing transitional care services for some patients (e.g., comprehensive discharge planning, health coaching support) [22], or care management follow-up based on each patient’s needs but not specifically tailored to the sepsis population; and when necessary receipt of ongoing care in post-acute skilled nursing facility or acute rehabilitation settings but with no sepsis-specific follow-up. To best align our pragmatic trial design with real-world conditions, aspects of Usual Care are determined by treating clinicians independent of trial assignment; however, we will actively surveil and document concurrent system-level interventions that may occur during the study period.

**Fig. 3:**
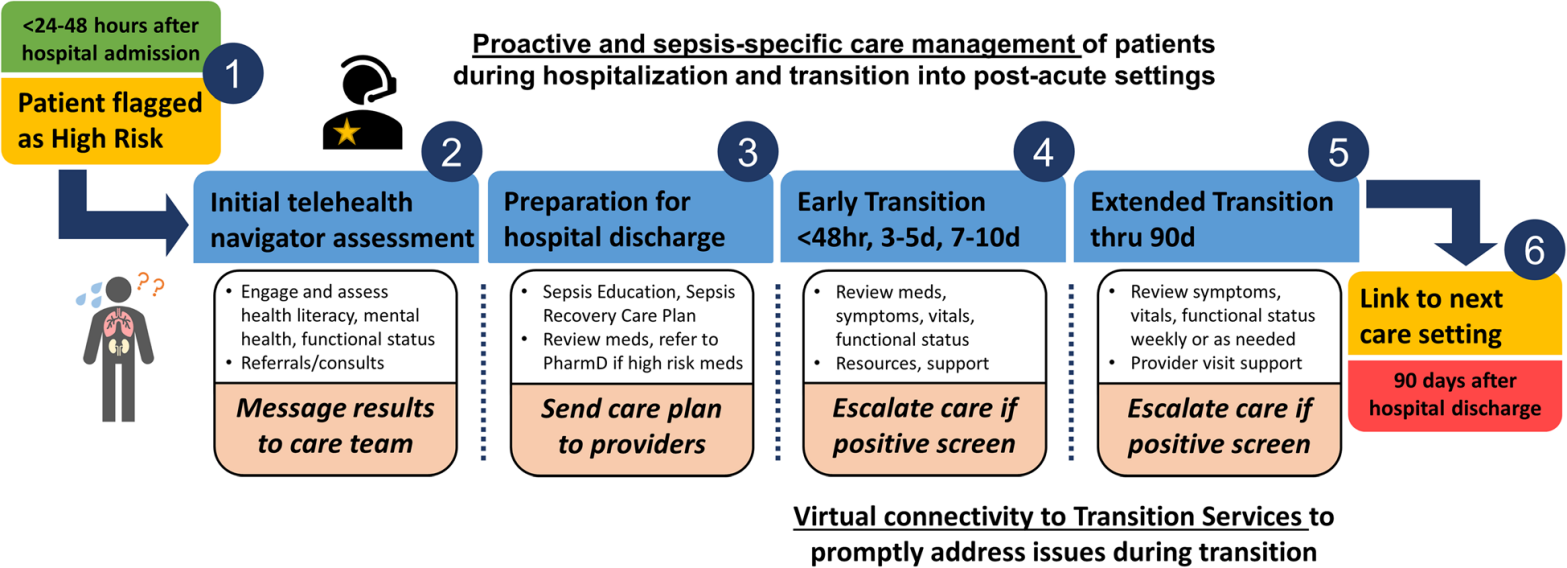
Sepsis Transition and Recovery (STAR) Program Description. The scheduled touchpoints for patients in the Sepsis Transition and Recovery (STAR) program are depicted. Patients and caregivers are first introduced to the STAR program during hospitalization at a participating Atrium Health (AH) facility. Specific STAR program tasks to be performed during the acute care (1, 2), discharge readiness (3), early post-acute transition (4), and 90-day post-acute (5, 6) intervals are summarized

Arm 2: Nurse Navigator-Driven STAR. Patients admitted to hospitals in the intervention arm at time of eligibility determination receive care via the STAR program (Fig. 3). The STAR program is described in detail elsewhere [49]. Broadly, STAR applies the Chronic Care Model organizational framework [28], which promotes care planning, active follow-up, and patient, provider, and community engagement, to increase adherence to best-practice recommendations and improve care coordination between hospital and post-acute care transitions during sepsis recovery. The STAR program employs a centrally-located nurse navigator who has clinical knowledge of sepsis and its cognitive and functional sequelae, core competencies in navigating transitions of care (e.g., facilitating communication, coordinating care, assessing/addressing barriers to care, providing patient education and practical resource information/referrals), and works as an extension of AH’s Transition Services within the Division of Hospital Medicine, which is a multi-disciplinary team providing acute care support during the peri-discharge interval [50]. The STAR navigator will provide proactive coordination and monitoring to patients using targeted, evidence-based best-practice care components: i) identification and treatment referral for new physical, mental, and cognitive deficits; ii) review and recommendation for adjustment of medications; iii) surveillance for treatable conditions that commonly lead to poor outcomes; and iv) referral to palliative care when appropriate (Table 2). In the current trial, the STAR navigator will provide telephone- and EHR-based support within the hospitalization and to patients across all discharge settings with remote follow-up at specified intervals throughout the 90 days post hospital discharge. Clinical oversight and care escalation (e.g., facilitation of community paramedicine in-home assessment, telemedicine video consult, in-person clinic visit) is supported by the Transition Services team and complementary to the patient’s primary care provider.

**Table 2:** Post-Sepsis Guidelines with Sepsis Transition and Recovery (STAR) Program Task

Core component / Evidence	Recommendation	STAR Task
**Screen for new physical, mental, and cognitive deficits after sepsis**		
Functional disability: Patients aged ≥65 years develop 1 to 2 new functional limitations	-Prescribe structured exercise program -Referral to Physical/ Cardiac/ Pulmonary rehab as needed	Confirm functional assessment (Physical Therapy). Refer as needed.
Swallowing impairment: Of patients aged ≥65 years, 1.8% readmitted < 90 days for aspiration pneumonitis	-Screen for cough, dysphagia, weak voice -Referral to speech therapy as needed	Confirm screen and team aware. Refer as needed.
Mental Health impairment: Prevalence for clinically significant anxiety 32%, depression 29%, and PTSD 44%	-Review details of hospital course (e.g., ICU diary) -Depression screen -Referral to peer support or Behavioral Health as needed	Mental health screen. Refer as needed.
**Review and Adjust Long-term Medications**		
Medication errors: Errors of omission and commission occur in up to 25% of patients, depending on medication	-Review antibiotic choice, dose, duration. -Start/continue meds for comorbidities; adjust for BMI, etc. -Discontinue hospital meds without ongoing indication	Antibiotic Stewardship Medication Reconciliation Vitals/Weight
**Anticipate and Mitigate risk for Common and Preventable Causes of Health Deterioration**		Routine virtual follow up. Schedule provider visits
Infection: Of patients aged ≥65 years, 11.9% readmitted < 90 days for infection (6.4% for sepsis)	-Patient education about symptoms of sepsis, recurrence -Appropriate vaccination -Monitor for symptomatic improvement in index infection	Education Medication Reconciliation Monitor symptoms
Heart failure exacerbation: Of patients aged ≥65 years, 5.5% readmitted < 90 days for CHF	-Reassess beta-blocker, diuretic, ACE-inhibitor dosing -Monitor volume status (fluid balance) - recognizing dry weight may be decreased if muscle mass lost	Medication Reconciliation Vitals/Weight Monitor symptoms
Acute Renal Failure: Of patients aged ≥65 years, 3.3% readmitted < 90 days for acute renal failure	-Monitor renal function; lab testing as needed -Reassess need and dosages for renally cleared, nephrotoxic agents	Monitor symptoms Confirm CBC/BMP Medication Reconciliation
COPD exacerbation: Of patients aged ≥65 years, 1.9% readmitted < 90 days for COPD exacerbation	-Confirm/initiate appropriate controller inhalers -Appropriate vaccination -Review use of benzodiazepines/opioids	Monitor symptoms Medication Reconciliation
**Assess appropriateness for palliative care**	-Palliative Care screen/consult as indicated -Goals of care. Educate on disease progression/ terminal	Discuss Palliative Care consult. Goals of Care

### Data collection

All clinical and outcomes data are collected directly from the AH EHR system and Enterprise Data Warehouse (EDW). Data are collected during the index hospitalization and include: basic sociodemographic characteristics (e.g., age, gender, race/ethnicity, marital status, insurance status at time of enrollment), past medical history (e.g., comorbidities, prior healthcare use), infection and disease severity (e.g., physiologic measurements [e.g., mean arterial pressure], lab values [e.g., complete blood count, basic/comprehensive metabolic panel, lactate]), hospital procedures (e.g., mechanical ventilation), and organizational variables (e.g., hospital location). Additional data are collected on care delivered during follow up and include: physical, speech, and occupational therapy, mental health assessment and referrals to behavioral health, outpatient follow-up visits, documented medication reconciliation in the EHR, Care Alignment Tool completion, and palliative care consultation. Navigators also document completion of STAR workflow processes in the patient’s EHR through the care management electronic documentation form, then data is exported into a research database (REDCap) [51].

### Study outcomes

The primary outcome is a dichotomous, composite measure of mortality and hospital readmission assessed 90 days post index hospital discharge. This combined outcome is ideally suited to our pragmatic study design because mortality and hospital readmission are widely regarded as patient-important outcomes [52]. In addition, patients’ risks for both mortality and hospital readmission remain elevated in the period following sepsis hospitalization [11, 53] and are uniformly captured from data contained in the AH EDW, minimizing non-differential assessment, outcome misclassification, and missing data. Moreover, preventing avoidable hospital readmission represents a critical quality metric for hospitals and has been targeted as a high priority for health care reform in the United States [54]. Finally, recent evidence from focused readmission reduction initiatives have shown decreased readmission rates [55] but also increased mortality during the same interval [56], highlighting the importance of measuring mortality and readmission rates in combination.

In ENCOMPASS, mortality is defined as any date of death documented in the AH EDW within 90 days of index hospital discharge, including events from national death record data uploaded monthly into the EDW via our institutional subscription [57]. Rehospitalization is defined as any inpatient or observation encounter to any of the more than 40 AH hospitals within the 90 days following index hospital discharge. Both inpatient and observation status rehospitalizations count towards a readmission because either status represents an adverse event important to patients and healthcare systems. In our primary analysis, mortality and hospital readmission events are weighted equally, and patients who experience either mortality or hospital readmission outcomes within 90 days of index hospital discharge are defined as event positive.

The following secondary clinical outcomes are assessed at 90 days after hospital discharge: 1) number of days alive and outside the hospital (i.e., a patient-centered, continuous measure of mortality and hospital readmission days that ranges from zero [most severe outcome] to 90 days [least severe outcome]) [58–60]; 2) all-cause mortality; 3) all-cause hospital readmission; 4) cause-specific hospital readmission with primary diagnoses (based on *International Classification of Diseases, 10th revision* diagnosis codes) related to: a) sepsis or common infection (i.e., sepsis [61], pneumonia [J13–18], urinary tract infection [N30, N34, N39.0], skin and soft tissue infection [L00–08]), b) chronic lung disease [J40–47], c) heart failure [I50], d) acute renal failure [N17], and e) the composite of ambulatory care sensitive conditions (as defined by the Centers for Medicare and Medicaid Services) [62]; 5) number of ED visits; and 6) number of outpatient visits.

### Primary statistical analysis

We will use an intent-to-treat approach to primary and secondary analyses, such that all patients meeting identical criteria and randomized will be analyzed, regardless of adherence to intervention assignment. This real-world approach will assess intervention effectiveness while limiting selection biases associated with adherence. Because hospitals are randomized to STAR in a staggered sequence and the outcome varies at the patient level, we will use a generalized linear mixed-effects model to compare the composite 90-day mortality and hospital readmission primary outcome measure between the intervention conditions [63]. The intervention fixed-effect coefficient will compare STAR versus Usual Care conditions (i.e., Usual Care as reference). We will include fixed effects for hospital cluster and time (i.e., month of enrollment) to adjust for hierarchical data structures and potential confounding due to secular trends and increased STAR program enrollment over time [63, 64]. Due to the stepped wedge design, there is potential for imbalance in patient- and hospital-level characteristics between study arms. We will include pre-specified covariates for patient characteristics at time of enrollment (e.g., age, sex, race, comorbidity score, disease severity) and organizational factors (e.g., hospital location) that may be related to mortality and hospital readmission in our adjusted model for primary analysis. Further, we will test for potential modification of the STAR treatment effect by conducting analyses stratified by pre-specified patient characteristics (e.g., age, comorbidity score, disease severity at trial enrollment) [65–67]. We will also extend our model framework to include interaction terms to assess: 1) treatment effect differences across hospitals; and 2) time effect differences across hospitals. In addition to primary analyses, we will conduct modified intent-to-treat analyses excluding sepsis patients 1) who do not survive index hospitalization since STAR is designed to support patients during their transition out of the hospital; and 2) discharged against medical advice since providers do not have the opportunity to deliver full care and prepare the patient for discharge [68]. Based on published data and internal estimates, we expect less than 6% of study patients will die during index hospitalization and less than 2% will be discharged against medical advice. We will present group comparisons as odds ratios and 95% confidence intervals. Similar to our primary analysis approach, we will construct generalized linear mixed-effects models for individual assessments of secondary outcomes. We will test different distribution parameters to determine the optimal distribution family for each model and outcome variable (e.g., binomial, negative binomial, gamma distributions). All hypothesis tests will be two sided and data will be analyzed using SAS (Cary, NC) or R (Vienna, Austria).

Methods to Handle Missing Data. We do not anticipate substantial missing data because all outcomes are routinely collected variables and utilization is broadly captured within our large integrated system. Values for patients who do not have health care utilization or mortality records during the study follow-up interval are assumed to be null. While utilization may occur outside AH, this is not expected to be a major limitation because of AH market share and accessibility. Specifically, AH operates three large hospitals in Cabarrus and Mecklenburg Counties, the only acute care hospitals in Anson, Burke, Cleveland, Lincoln, Stanly, and Union counties, where most of this study will be conducted, and more than 40 hospitals in the region overall. Additionally, any utilization occurring outside the system is anticipated to be non-differentially distributed between groups and thus impact treatment groups equally. Further, internal historical data indicates nearly 75% of high-risk patients are Medicare-insured (i.e., Medicare Shared Savings Plan beneficiaries). For these patients, we will have complete healthcare claims within and outside AH facilities during the study interval, as captured through participation in the local AH-managed Accountable Care Organization. We will conduct subgroup analyses within this Medicare-insured population and will use this data to explore missing data patterns that can be adjusted using pattern-mixture methods in sensitivity analyses [69, 70].

### Sample size and statistical power

Based on our internal data from sepsis admissions to the study hospitals between 2015 and 2017, we estimate a total of 4480 patients will be eligible for participation during the 36-month ENCOMPASS trial enrollment period (i.e., approximately 16 high risk patients per hospital per month). From this historical data, we observed a 90-day mortality rate of 18% and 90-day hospital readmission rate of 42%. From published literature, a small percentage of deaths (~ 1–2%) and between 22 and 42% of hospital readmissions after sepsis are preventable [10, 71, 72]. Using a stepped-wedge design [64, 73] with eight hospitals and intra-cluster correlation coefficients ρ = 0.1 to account for the hierarchical data structure and potential clustering of intervention effects within hospitals, nine time periods (including baseline), eight steps with one hospital switching from UC to STAR at each step, and total sample size of at least 4032 patients enrolled over 36 months (i.e., at least 90% of historical patient volumes), we will have 90% power (α = 0.05) to detect a 8% absolute change in the composite 90-day mortality and hospital readmission outcome that is important to patients and clinically meaningful.

### Implementation evaluation

Outcome measures for evaluation of the STAR program implementation are summarized in Table 3. We will use baseline assessments of implementation climate and ongoing evaluation of STAR implementation to inform and adapt its execution. Mixed-methods data will be collected through surveys, qualitative semi-structured interviews, and focused ethnography. We will use the CFIR to guide development of data collection, coding, and data analysis [35, 36]. The CFIR provides a standardized taxonomy of operationally defined constructs culled from multiple disciplines relevant to implementation of complex programs. We will apply five CFIR domains: intervention characteristics (e.g., evidence strength and quality, relative advantage, adaptability, trialability, complexity); outer setting (e.g., needs and resources of sepsis patients, cosmopolitanism, healthcare system policies and incentives); inner setting (e.g., hospital and healthcare system networks/communication, hospital and healthcare system implementation climate, readiness); characteristics of individuals (e.g., knowledge and beliefs); and process used to implement the program (e.g., quality and extent of planning, engagement of key stakeholders, evaluation). We will also include elements from the Reach, Effectiveness, Adoption, Implementation, and Maintenance (RE-AIM) [74] model to quantify the reach, effectiveness, and costs of the STAR program and its potential for dissemination and scalability.

**Table 3:** Summary of Outcome Measures for Evaluation of STAR Program Implementation

			Implementation time point				
	Assessment	Evaluation tool	Pre	8 m	20 m	32 m	Post
**CFIR Construct Assessed**							
Outer setting (Patient Needs and resources, Cosmopolitanism, external policies/incentives)	Administrative leaders	CFIR interviews	X				X
	Providers	CFIR interviews	X				X
Inner Setting (networks/communication, implementation climate, readiness)	Administrative leaders	CFIR interviews	X				X
	Providers	CFIR interviews	X	X	X	X	X
	Navigators	CFIR interviews		X	X	X	
Intervention (evidence strength/quality, relative advantage, adaptability, trialability, complexity)	Administration	CFIR Interviews		X	X	X	
	Providers	CFIR Interviews		X	X	X	
	Navigators	CFIR Interviews		X	X	X	
Individuals (knowledge and beliefs about intervention, self-efficacy)	Providers	Knowledge survey Self-Efficacy survey		X	X	X	
	Navigators	Knowledge survey Self-Efficacy survey		X	X	X	
	Patients and Caregivers	Knowledge survey Self-Efficacy survey		X	X	X	
Process (execution)	Navigators	Focused ethnography		X	X	X	
**Other outcomes assessed**							
Reach, Adoption, and Maintenance	Patients (Reach)	1) Navigator use per eligible patients 2) risk prediction for those enrolled		X	X	X	X
	Providers (Adoption)	# of providers with pts. enrolled		X	X	X	X
Effectiveness	Healthcare utilization	90-day mortality or readmission	X				X
Cost	Health system	Clinical trial data					X
	Societal	Extrapolated					X

*CFIR* Consolidated Framework for Implementation Research

We will collect data before, during, and after implementation. For pre- and post-implementation data collection, we will conduct qualitative interviews with eight administrative leaders and eight providers regarding organizational support, culture, and recommendations for STAR implementation and then post-implementation to plan future dissemination. During the implementation phase, we will gather data at three time points (i.e., 8-, 20-, and 32-months) from: a) qualitative interviews and surveys with navigators and providers to assess and understand barriers to and enablers of STAR implementation and to help improve access to sepsis care, b) qualitative interviews and surveys with patients and caregivers (recruited from intervention arms) to learn about their experiences with STAR during the peri-discharge transition, opinions and perspectives on services received and not received, and preferences for care, and c) focused ethnography of the navigators to obtain a holistic and nuanced understanding of the navigator’s role in the STAR intervention [75, 76]. Interview recordings and ethnographic observation fieldnotes will be transcribed for coding and qualitative analysis using ATLAS.ti v8.0. We will use both inductive and deductive coding strategies guided by the CFIR and constant comparison to perform content analysis, applying iterative comparison of newly coded text with previously coded text of the same theme until final thematic refinement is achieved [77]. We will finalize the code list and repeat data review using the finalized code structure for coding reliability.

To increase policy impact, we will also conduct a prospective economic evaluation to provide an understanding of the resource implications of the STAR program intervention on post-sepsis health benefits and costs. We will combine primary data and mathematical modeling to examine the costs and cost-effectiveness of STAR over the 90-day study follow up as a measure of benefit from the health system perspective [78]. We will also incorporate evidence-based extrapolations of selected post-sepsis related risk factors, future disease burden, and Markov-type state transition simulation modeling to project cost-effectiveness 12 months after hospital discharge as a measure of benefit from the societal perspective [79–83]. The investigation will collect detailed information on costs associated with the addition of STAR program services (e.g., dedicated program staff - nurse navigators, training) plus the total variable costs of healthcare services received over the 90-day study follow-up period. Quality Adjusted Life Years will be estimated based on utility assessments from published literature [84–86] adjusted for factors at hospital discharge (e.g., ICU days, age, comorbidities), subsequent rehospitalization, and mortality. In both 90-day and 12-month approaches, we will assess the incremental cost effectiveness ratio defined as incremental change in costs divided by incremental change in effectiveness. These data will provide benchmarks to support resource allocation for STAR program scale-up and spread and inform health policy decisions related to post-sepsis care [87].

### Data and safety monitoring

The ENCOMPASS trial is overseen by a Data and Safety Monitoring Board (DSMB) comprising experts in critical care, infectious disease, internal medicine, biostatistics, and epidemiology. The DSMB and study investigators agreed not to establish formal guidelines for stopping but are monitoring 90-day all-cause mortality rates and major depressive disorder using the Patient Health Questionnaire-9 [88] as safety endpoints. Due to the low risk associated with study participation, there are no planned interim analyses of the primary outcome.

## Discussion

Healthcare systems urgently need programs to better support sepsis survivors following hospital discharge —a key priority that has been intensified as a result of the coronavirus disease 2019 pandemic and anticipated increasing number of individuals impacted by sepsis [89]. The ENCOMPASS stepped-wedge hybrid type I effectiveness-implementation trial advances knowledge of implementation and delivery of post-sepsis care in multiple ways. First, despite the societal burden and cost associated with sepsis, there is limited evidence to guide management of patients after hospitalization for sepsis. The STAR program is a telehealth nurse navigator-led post-sepsis transition intervention that combines multiple evidence-based components for application in a heterogenous healthcare setting. STAR and its evaluation have been designed with elements to enhance generalizability of results (e.g., diverse study hospitals, expert care recommendations, international guidelines for clinical identification of sepsis patients) and sustainability and scalability to multiple settings locally, regionally, and nationally (e.g., resource-conservative, virtually connected navigators). For example, using telehealth to deliver post-sepsis care elements overcomes the barriers identified in the literature concerning accessibility and reach of clinic-based follow-up care for survivors of critical illness [90]. Further, the telehealth platform extends the reach of nurse navigators across a geographically diverse cohort.

Second, effectiveness studies for complex healthcare problems like this one too often neglect the vital importance of evaluating and adapting implementation practices based on robust implementation science frameworks and constructs. Similarly, there has been no dissemination and implementation research specifically on post-sepsis care in the context of a determinants framework to establish effective processes and their outcomes. Our proposal is innovative in its emphasis on optimization of dissemination and implementation of post-sepsis care in heterogenous healthcare settings, while enhancing traditional implementation frameworks with focused ethnography to obtain a more nuanced understanding of the nurse navigator’s role in implementation success. By evaluating the implementation and effectiveness of the STAR intervention across eight diverse hospitals, we will generate knowledge that will immediately be relevant for a broad range of acute care settings and healthcare systems. Additionally, incorporating cost effectiveness analyses will further provide key information to stakeholders to guide future use and scale-up of the STAR program, if shown to be effective and cost-favorable. Thus, the proposed project has the potential to shift the paradigm of how post-sepsis care can be more effectively implemented and disseminate the information widely to improve care and costs for millions of sepsis survivors.

## Supplementary Information

**Additional file 1.**

## Data Availability

Datasets generated during the current study will not be shared, consistent with data governance and policies.

## Abbreviations

AH: Atrium Health
CFIR: Consolidated Framework for Implementation Research
DSMB: Data and Safety Monitoring Board
EDW: Enterprise Data Warehouse
EHR: Electronic Health Record
ENCOMPASS: Engagement and Collaborative Management to Proactively Advance Sepsis Survivorship
PRECIS-2: Pragmatic Explanatory Continuum Indicator Summary 2
RE-AIM: Reach, Effectiveness, Adoption, Implementation, and Maintenance
SOFA: Sequential Organ Failure Assessment
SPIRIT: Standardized Protocol Items: Recommendations for Interventional Trials
STAR: Sepsis Transition and Recovery
